# Laboratory characteristics analysis of the efficacy of levothyroxine on subclinical hypothyroidism during pregnancy: a single-center retrospective study

**DOI:** 10.1080/21655979.2021.1955589

**Published:** 2021-07-21

**Authors:** Luyang Han, Yan Ma, Zhaoxia Liang, Danqing Chen

**Affiliations:** Department of Obstetrics, Women’s Hospital School of Medicine Zhejiang University, Hangzhou, China

**Keywords:** Hypothyroidism, pregnancy outcome, lipoproteins, homocysteine, levothyroxine

## Abstract

To reassess the efficacy of levothyroxine on subclinical hypothyroidism (SCH, 4.0 mIU/L ≤ TSH (thyroid stimulating hormone) <10 mIU/L with normal free T4) during pregnancy. 165 levothyroxine-treated pregnant women experiencing SCH were screened. And controls were randomly selected using euthyroidism (EU) women, matched by age, gravidity, and parity in the EU group (*n* = 660). We evaluated laboratory characteristics and pregnancy outcomes during follow-ups. Compared with the EU group, the SCH group displayed higher inadequate maternal gestational weight gain, premature delivery, low birth weight offspring and infant offspring small for their gestational age. After levothyroxine treatment, the SCH group displayed lower total cholesterol, low-density lipoprotein levels, and higher serum homocysteine levels before delivery. Pregnant women with SCH still exhibit adverse pregnancy outcomes after levothyroxine treatment. Taken together, we believe that besides levothyroxine, vitamin B12 and folic acid could be added to the treatment of pregnant women with SCH. In addition, regular monitoring of blood sugar levels, lipid and homocysteine levels, and intervention gestational weight gain could alleviate the adverse effects of SCH on pregnancy outcomes.

## Graphical Abstract

**Figure:**
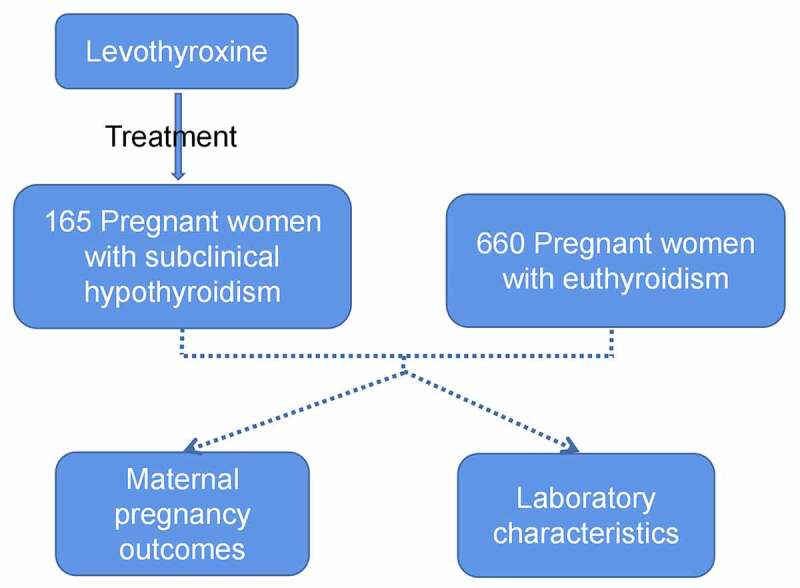

## Introduction

The incidence of subclinical hypothyroidism (SCH) in pregnancy ranges 2%–15% [1]. SCH is defined as elevated serum thyroid stimulating hormone (TSH) levels and normal serum thyroxine (T4) levels [2]. The diagnostic criteria for SCH in pregnancy have changed over the years, with varying TSH thresholds ranging from 2.5 mU/L [3] to 4 mU/L [4]. Several studies have demonstrated the association between gestational SCH and adverse pregnancy outcomes, including miscarriage, early eclampsia, placental abruption, preterm birth, and low birth weight (LBW) [1,5–9]. Hence, the impact of SCH on the pregnancy outcome merit further investigation.

Currently, levothyroxine has been recognized as the most effective and convenient drug for the treatment of SCH, reducing the risk of adverse pregnancy outcomes [10]. Rahman et al. [11] demonstrated that levothyroxine increased embryo implantation and live birth rates, and decreased miscarriage rates. A randomized controlled trial (RCT) by Rao et al. [10] found that levothyroxine reduced the rate of miscarriage, but no effect on clinical pregnancy rates was observed. In addition, Yamamoto et al. [12] similarly found no significant difference in any clinical pregnancy outcome in levothyroxine treated SCH patients during pregnancy compared to the untreated group. All of these studies suggest that it remains unclear whether the use of levothyroxine improves pregnancy outcomes in women with SCH. Therefore, we set up a RCT to investigate the impact of levothyroxine treatment on pregnancy outcomes in women with SCH. Besides, by analyzing the biochemical indices of pregnant women in the middle and late stages of pregnancy, we determined the differences in pregnancy outcomes between the SCH and euthyroidism (EU) groups under levothyroxine treatment and improved the treatment of SCH with the aim of providing new ideas for clinicians.

## Materials and methods

### Study cohort

We conducted a single-center retrospective study on pregnant women with a single fetus from the second trimester until delivery. All pregnant women received prenatal care at Zhejiang University Affiliated Maternity Hospital (Hangzhou, China) (*n* = 14,123). Of note, we excluded women with a known medical history (*n* = 2121), including pre-pregnancy thyroid disease, chronic hypertension, diabetes, autoimmune disease, mental illness, or other major diseases like cardiovascular diseases, from the study. Of all patients (*n* = 12,002), 165 women were diagnosed with SCH (SCH group) (4.0 mIU/L ≤ TSH < 10 mIU/L with normal FT4). All patients received levothyroxine treatment during pregnancy as prescribed. The initial dosage depended on the serum TSH level of the patients. The thyroid function of patients was tested every 4 weeks, and the drug dosage was adjusted according to their serum TSH level until delivery. Owing to the hospital treatment policy, there were no untreated SCH patients. We randomly selected controls as women with EU (0.2 ≤ TSH < 4.0 mIU/L; EU group) who had never received levothyroxine treatment. Each control case was similarly matched with four controls regarding age, gravidity, and parity. We enrolled 660 controls in this study. This study protocol was approved by the constituted Ethics Committee of Women’s Hospital, School of Medicine, Zhejiang University.

### Data collection

We collected baseline characteristics in the second trimester when patients first visited our hospital. The current Chinese policy encourages pregnant women to conduct early antenatal checkup (before 24 weeks) at community health service organizations. Thus, early pregnancy data were not completely available, except for progestational body mass index (BMI). The levels of TH, cholesterol, and blood sugar were measured in both the second and third trimesters of pregnancy. Of note, the third-trimester data obtained from the prenatal examination are the most recent data before delivery. In addition, we obtained biparietal diameter and femur lengths from the latest prenatal B-mode ultrasound before delivery; these values were measured and recorded by professional obstetricians. Next, composite maternal outcomes, were compared. We used EpiData 3.1 for data entry.

### Statistical analysis

All statistical analyses in this study were performed using IBM SPSS Statistics 20 for Windows (Stata Corp., College Station, TX). One-sample Kolmogorov–Smirnov test (K–S test) was adopted initially to identity the normal distribution of continuous data. Non-normally distributed variables, presented as the median interquartile range (IQR), were compared using the Mann–Whitney *U*-test. All categorical variables are presented as frequencies (percentages); these were contrasted using $\chi^{2}$ tests. In addition, we performed binary regression analyses (forward, LR) to investigate independent factors related to pregnant women with SCH. Of note, the results of paired regressions are presented as odds ratios (ORs) and 95% confidence intervals (CIs). In this study, we considered *P*< 0.05 as statistically significant.

## Results

We collected pregnant women with SCH treated with levothyroxine and those in the EU group and compared pregnancy outcomes and laboratory indicators between the two groups to determine the effect of levothyroxine treatment on pregnancy outcomes in women with SCH.

No statistically significant differences were observed in the demographic data between the EU and SCH groups (Table 1). The SCH group exhibited a higher median [IQR] serum TSH level compared with the EU group (4.54 [IQR: 4.20–4.99] mIU/L vs. 1.42 [IQR: 1.02–1.96] mIU/L; *P* < 0.0001). Besides TSH, the median levels of total thyroid (TT4), total triiodothyronine (TT3), free thyroid (FT4), and free triiodothyronine (FT3) were not significantly different between both groups (data not shown).

**Table 1.** General characteristics of pregnant women with subclinical hypothyroidism (SCH) and euthyroidism (EU)

Clinical characteristics	Group SCH, (n = 165)	Group EU, (n = 660)	p-Value
TSH, median (IQR^a^), mIU/L	4.54 (4.20, 4.99)	1.42 (1.02, 1.96)	.000*
Gestational age at the time of TSH testing, median (IQR), weeks	25.29 (23.57, 26.64)	25.14 (23.86, 26.57)	.152
TSH antibody +, n (%)	0	39 (23.64)	-
Thyroglobulin antibody +, n (%)	0	27 (16.36)	-
Thyroid peroxidase antibody +, n (%)	0	29 (17.58)	-
Maternal age,median (IQR), yrs	29 (26 32)	29 (26.32)	1.000
Primipara, n (%)	315 (47.73)	78 (47.27)	.950
History of abortion (at least two losses)	85 (12.88)	21 (12.73)	.964
Progestation BMI^b^, median (IQR), kg/m^2^	20.01 (18.63, 21.64)	20.20 (18.25, 21.88)	.750
Underweight, n (%)	150 (22.73)	48 (29.09)	.087
Normal weight, n (%)	455 (68.94)	106 (64.24)	.247
Overweight or Obesity, n (%)	35 (5.15)	9 (4.85)	.938
Smoker /Alcohol or illicit drug, n (%)	1 (0.15)	0	-
Assisted reproduction, n (%)	7 (1.06)	1 (0.6)	-

^a^TSH: Thyroid stimulating hormone; IQR: Interquartile range.

^b^Progestation BMI: Progestation body mass index, was calculated using reported height and progestation weight and categorized according to World Health Organization (WHO). Categories: underweight: <18.5 kg/m^2^; normal: 18.5 ≤ *x* < 24 kg/m^2^; overweight: 24 ≤ *x* < 28 kg/m^2^; obese ≥28 kg/m^2^

Regarding maternal pregnancy outcomes, the SCH group displayed a higher preterm delivery rate compared with the EU group (9.1% vs. 4.4%; *P*= 0.016). Specifically, SCH correlated with an increased risk of premature delivery at <37 weeks; however, it did not correlate with premature delivery at <34 weeks or <36 weeks. In addition, the median [IQR] of gestational weight gain (GWG) in the SCH group was significantly lower than that in the EU group (14.0 [11.0–16.0] vs. 15.0 [12.0–17.0]; *P* = 0.003). Based on the American College of Obstetricians and Gynecologists recommended weight gain during pregnancy [13], inadequate GWG women in the SCH group were more than that in the EU group (25.77% vs. 18.59%; *P* = 0.041; OR 0.599; 95% CI: 0.402–0.892). Besides, the EU group had more GWG pregnant women than that in the SCH group (*P* = 0.001). We observed no significant differences between both groups regarding gestational diabetes, postpartum hemorrhage, or abortions. For neonatal pregnancy outcomes, the SCH group reported a higher number of LBW infants (5.45% vs. 1.97%) and infants small for their gestational age (SGA; 4.24% vs. 0.60%) compared with the EU group (*P* < 0.05). Moreover, the median lengths of newborns and the biparietal diameters measured by prenatal ultrasound were significantly less than those in the EU group (*P* < 0.05). We observed no significant differences in birth weight, sex ratio, and malformation rate. Furthermore, no significant differences were found in neonatal admission rate, average length of stay, and reasons for hospitalization between both groups (Table 2).

**Table 2.** The perinatal outcomes of pregnant women with subclinical hypothyroidism (SCH) and euthyroidism (EU)

Maternal outcomes	Group SCH, n = 165	Group EU, n = 660	p-Value	OR	95%CI
Gestational weeks, median (IQR), weeks	39.43 (38.57, 40.29)	39.57 (38.71, 40.50)	.855		
GWG^a^, median (IQR), kg	14.0 (11.0, 16.0)	15.0 (12.0, 17.0)	.003	1.066	(1.109–116)
Appropriate	84 (51.54)	290 (45.31)	.155		
Inadequate	42 (25.77)	119 (18.59)	.041	.599	(0.402–892)
Excessive	37 (22.70)	231 (36.09)	.001	1.187	(1.037–1.358)
Premature delivery, n (%)					
<34 weeks, n (%)	2 (1.21)	2 (0.30)	.180		
<36 weeks, n (%)	6 (3.64)	14 (2.12)	.260		
<37 weeks, n (%)	15 (9.09)	29 (4.39)	.016	.460	(0.240–0.879)
Cesarean section delivery, n (%)	69 (41.82)	228 (34.55)	.082		
PIHs^b^, n (%)	9 (5.45)	21 (3.18)	.163		
Gestational hypertension, n (%)	6 (3.63)	16 (2.42)	.387		
Mild preeclampsia, n (%)	0	6 (0.91)	-		
Severe preeclampsia, n (%)	3 (1.81)	0	-		
GDM^c^, n (%)	25 (15.15)	82 (12.42)	.351		
Postpartum hemorrhage, n (%)	1 (0.61)	4 (0.61)	1.000		
Abortion (<28 w), n (%)	0	0	-		
Polyhydramnios, n (%)	1 (0.61)	12 (1.82)	.483		
Oligoamnios, n (%)	6 (3.64)	16 (2.42)	.384		
PROM^d^, n (%)	36 (21.82)	133 (20.15)	.635		
Neonatal outcome					
Neonatal weight, median (IQR), g	3300 (2950, 3550)	3300 (3000 3600)	.245		
HFD^e^, n (%)	9 (5.45)	33 (5.0)	.812		
LBW^e^, n (%)	9 (5.45)	13 (1.97)	.026	.240	(0.094–0.614)
SGA^e^, n (%)	7 (4.24)	4 (0.60)	.002	.069	(0.014–0.333)
Body length (IQR)^f^, cm	50.0 (50.0, 50.0)	50.0 (50.0, 50.0)	.034	1.144	(0.997–1.312)
BPD, median (IQR), cm	9.20 (9.00, 9.50)	9.30 (9.10, 9.50)	.025	1.864	(1.231–2.823)
FL,median (IQR), cm	7.00 (6.90, 7.20)	7.10 (6.90, 7.30)	.174		
Low Apgar score (<7 at 1 minutes), n (%)	3 (1.82)	8 (1.21)	.544		
Malformation, n (%)	1 (0.61)	6 (0.91)	.704		
Neonatal admission, n (%)	50 (30.30)	181 (27.42)	.444		
Neonatal hyperbilirubinemia, n (%)	23 (13.94)	90 (13.64)	.919		
Respiratory causes^g^, n (%)	11 (6.67)	25 (3.79)	.105		
Blood glucose disorder^g^, n (%)	3 (1.82)	21 (3.18)	.351		
Neonatal admission, median (IQR), days	0.0 (0.0, 4.0)	0.0 (0.0, 4.0)	.204		

^a^GWG, gestational weight gain; appropriate, inadequate, and excessive were according to [13] (Table 1).

^b^PIHs: pregnancy-induced hypertension syndrome.

^c^GDM, Gestational Diabetes Mellitus.

^d^PROM, Premature rupture of membranes.

^e^LFD, light for date, neonatal weight less than 2500 g; HFD, heavy for date, neonatal weight greater than 4000 g.

SGA, small for gestational age infant.

^f^The average (SD) of neonatal body length in the SCH and EU groups were 49.60 ± 1.41 and 49.85 ± 1.07, respectively.

^g^Respiratory causes: acute respiratory distress syndrome, neonatal asphyxia. Blood glucose disorder: hyperglycemia or hypoglycemia.

.

In the second trimester, high-density lipoprotein (HDL, *P* = 0.014) and LDL (*P =* 0.005) in the SCH group were significantly lower than that in the EU group; these same phenomena can be observed in the third trimester. Regarding blood glucose, the results of the oral glucose tolerance test indicated no significant differences between both groups. However, the glycosylated hemoglobin Alc (HbA1c) levels in the SCH group during the second trimester were marginally but significantly lower than that in the EU group (4.90 [4.70–5.10] vs. 4.90 [4.70–5.07]; *P* = 0.007). Moreover, the fasting blood glucose and the glycosylated albumin in the SCH group were lower than that of the EU group during the third trimester. Furthermore, the Hcy concentration in the SCH group (6.20 [5.10–7.73]) was significantly higher than that in the EU group before delivery (5.40 [4.60–6.50], *P* = 0.000; Table 3).

**Table 3.** Comparison of laboratory characteristics between pregnant women with subclinical hypothyroidism (SCH) and euthyroidism (EU)

*Index, median (IQR)*	Group SCH, n = 165	Group EU, n = 660	p-Value	OR	95%CI
***During the second trimester***					
Gestational age at the time of testing, weeks	25.14 (23.86 26.57)	25.29 (23.57 26.64)	.152		
TC^a^, mg/dL	6.10 (5.53, 6.73)	6.20 (5.57, 6.82)	.161		
TG^a^, mg/dL	1.92 (1.60, 2.51)	2.06 (1.67, 2.60)	.253		
HDL^a^, mg/dL	2.21 (1.93, 2.57)	2.37 (2.07, 2.80)	.014	1.734	(1.215–2.474)^d^
LDL^a^, mg/dL	3.20 (2.59, 3.82)	3.40 (2.88, 3.92)	.005	1.311	(1.060–1.622)^d^
FBG^a^, mmol/L	4.43 (4.23, 4.65)	4.45 (4.24, 4.69)	.871		
Pbg1h, mmol/L	7.45 (6.30, 8.83)	7.75 (6.76, 8.80)	.094		
Pbg2h, mmol/L	6.35 (5.67, 7.41)	6.61 (5.80, 7.51)	.513		
HbA1c, %	4.90 (4.70, 5.10)	4.90 (4.70, 5.07)	.007		
***During the third trimester***					
Gestational age at the time of testing, weeks	39.57 (37.57, 41.14)	39.00 (37.14, 40.57)	.055		
TC, mg/dL	6.26 (5.49, 7.06)	6.54 (5.80, 7.47)	.029	1.176	(1.039–1.332)^d^
TG, mg/dL	3.14 (2.38, 4.05)	2.87 (2.22, 3.70)	.961		
HDL, mg/dL	1.93 (1.69, 2.26)	2.08 (1.79, 2.42)	.015	1.102	(0.895–1.356)^d^
LDL, mg/dL	3.34 (2.73, 4.03)	3.61 (3.03, 4.27)	.004	1.440	(1.076–1.927)^d^
FBG, mmol/L	4.64 (3.98, 5.48)	4.97 (4.30, 5.76)	.008		
Serum glycated albumin, mg/dL	11.90 (11.30, 12.60)	11.85 (11.20, 12.60)	.012		
homocysteine, mg/dL.	6.20 (5.10, 7.73)^b^	5.40 (4.60, 6.50)^b^	.000	.710	(0.595–0.846)^d^
FT4, mIU/L	11.83 (9.9,12.70)^c^	11.50 (10.60, 12.40)^c^	.682		
TSH, mIU/L	2.77 (1.87, 4.11)^b^	1.68 (1.21, 2.50)^b^	.004	.548	(0.442–0.680)^d^

^a^TC: Total cholestrol; TG: Triglycerides, HDL: high density lipoprotein; LDL: Low Density Lipoprotein; FBG: fasting blood glucose.

^b^Group SCH, n = 138; Group EU, n = 576.

^c^Group SCH, n = 96; Group EU, n = 334.

^d^Adjusted for age, gestational age, progestational body mass index (BMI), and infant sex.

## Discussion

For now, the diagnosis and treatment of SCH in pregnant women remains controversial. And even a mild maternal thyroid hormone deficiency has a negative impact on pregnancy outcome and offspring mental development [14]. Premature delivery and LBW are two common adverse pregnancy outcomes in pregnant women with SCH [4]. León *et al*. reported that for every SD increase in TSH, birth weight decreased by 19 g (95% CI: −36 to −2) [15]. Compared with the untreated SCH group, Maraka *et al*. reported that levothyroxine-treated pregnant women with SCH exhibited a decreased risk of LBW (1.3% vs. 10%; *P*  <  0.001) [16]. Our study demonstrated that SCH did not affect neonatal weight after treatment; however, the prevalence of LBW fetuses in the SCH group remained higher than that in the EU group, even posttreatment. Meanwhile, we also observed that the number of women in the SCH group who did not gain sufficient weight during pregnancy was significantly higher than that in the EU group. Reportedly, inadequate GWG increases the risk of premature delivery, LBW, and SGA [17,18]. Furthermore, ensuring the GWG growth within a reasonable range for SCH patients could reduce the birth of children with LBW and SGA.

We found that even with levothyroxine treatment, the occurrence of preterm delivery in the SCH group was markedly higher than that in the EU group; however, this difference only existed when the gestational age was <37 weeks. We observed no significant difference in the number of preterm deliveries between both groups at the gestational age of <32, 34, or 36 weeks. In addition, no difference was noted in the pregnancy duration between both groups. Casey *et al*. reported that compared with untreated pregnancy or the EU group, the presence of SCH correlated with premature delivery at <34 weeks; however, this did not increase the risk of premature delivery at <32 or at <36 weeks [19]. Moreover, Nazarpour *et al*. suggested that levothyroxine treatment could precisely decrease SCH by adhering to the newly recommended cutoff of ≥4.0 mIU/L [20]. Our study found that TH supplementation could improve the frequency of premature delivery caused by SCH; however, the adverse outcomes persist after TH supplementation, suggesting that this treatment method might require further improvement.

Imbalances in glycolipid metabolism are associated with various metabolic syndromes, which may eventually lead to adverse pregnancy outcomes for fetuses. We noted a difference in HBA1c during the second trimester (*P* = 0.007), as well as in glycosylated albumin (*P* = 0.012) and fasting glucose (*P* = 0.008) during the third trimester. These findings corroborated previous studies that suggested that glycometabolism was significantly different between patients with EU and SCH even after levothyroxine treatment [21]. Besides, HDL and LDL in the second and third trimesters of pregnancy in the SCH group were significantly lower than that in the EU group. Our findings corroborate a recent study that demonstrated that T4 replacement therapy could decrease TC and LDL [22].

In addition, we found that Hcy levels in the SCH group were markedly higher than those in the EU group. Some previous studies have demonstrated that high Hcy levels in mothers correlated with PIH, premature delivery, intrauterine growth retardation [23–25]. Yajnik *et al*. found that offspring birth weight inversely correlated with the maternal Hcy concentration at −40 g/SD (95% CI: −62 to −17); besides, a one SD increase in the plasma Hcy concentration estimated a 0.1-week earlier delivery [26]. Reportedly, Hcy could promote the expression of chemokines and insulin resistance by inducing endoplasmic reticulum stress in patients with excess adipose tissue and hypothyroidism [27]. THs could interfere with Hcy metabolism by stimulating the processes of vitamin B12- and folic acid-dependence and affecting enzymes in the methylation pathways [28]. Moreover, levothyroxine treatment could result in a significant decrease in Hcy levels. Supplementation with methylfolate, vitamin B6, and vitamin B12 has been demonstrated to be effective for lowering Hcy levels and improving pregnancy outcomes [29]. We believe that vitamin B12, B6, and folic acid supplementation, combined with the monitoring of glycolipid changes and Hcy levels, could also help women with SCH attain a more desirable pregnancy outcome; however, reliable evidence supporting the idea that pregnant women with SCH will experience more desirable outcomes in response to this therapy is required.

This study has some limitations worth acknowledging. First, it is a single-center retrospective study, and the information was obtained primarily by reviewing medical records; this could have resulted in a degree of information bias. Second, patients were not strictly monitored for medications. Although we adjusted dosage according to the thyroid levels of pregnant women at every prenatal visit, the likelihood that medication dosages were missed is undeniable.

## Conclusions

This study reported that levothyroxine-treated patients with SCH (4.0 mIU/L ≤ TSH < 10 mIU/L) still had high incidences of adverse pregnancy outcomes, including inadequate GWG, premature delivery, LBW, infants SGA, and others. Hence, this study establishes that the current treatment method could be improved by vitamin B12 and folic acid supplementation. Furthermore, regular monitoring of blood sugar, lipids, and Hcy levels, in conjunction with GWG monitoring, could improve the adverse effects of SCH on pregnancy outcomes.

## Highlights

1. Reduction in total cholesterol and LDL levels before delivery in pregnant women with SCH after levothyroxine treatment.
2. Elevated serum homocysteine levels before delivery in pregnant women with SCH after levothyroxine treatment.
3. Pregnant women with SCH still exhibit adverse pregnancy outcomes after levothyroxine treatment.
